# Genetic Features of the Scuticociliate Pathogen *Philaster* sp. Isolate FWC2 That Causes Sea Urchin Mass Mortality

**DOI:** 10.1111/jeu.70065

**Published:** 2026-02-02

**Authors:** Shen Jean Lim, Mya Breitbart

**Affiliations:** ^1^ College of Marine Science University of South Florida St. Petersburg Florida USA

**Keywords:** ciliate, DaScPc, *Diadema*, mitochondria, mitogenome, scuticociliate, scuticociliatosis, urchin

## Abstract

A scuticociliate most closely related to *Philaster apodigitiformis* caused mass mortalities of diadematoid sea urchins and was cultured as *Philaster* sp. isolate FWC2. We sequenced the metagenomic content of this isolate, which was predicted to represent ≤ 56% of the complete genome. Based on *k*‐mer counts, the haploid genome size was predicted to be 122–136 Mbp. We assembled and annotated a 4,088 bp nuclear ribosomal operon, a 41,396 bp mitochondrial genome with 19.22% G + C content, 24 protein‐coding genes, 6 tRNA genes, and 2 rRNA genes, and a protein sequence homologous to *β‐PKA* in *Philaster apodigitiformis* potentially involved in host infection.

## 1 Introduction

Scuticociliates (phylum: Ciliophora, class: Oligohymenophorea, subclass: Scuticociliatia) are prevalent in marine ecosystems, where they serve important trophic roles in food webs (Pierce and Turner 1992). Some scuticociliates cause scuticociliatosis in fishes (Piazzon et al. 2013; Kim et al. 2025), urchins (Hewson et al. 2023; Zirler et al. 2023; Ritchie et al. 2024; Roth et al. 2024), and other marine organisms (Small et al. 2005). In 2022, a scuticociliate pathogen with ribosomal RNA (rRNA) gene sequences most closely related to *Philaster apodigitiformis* caused mass mortality of the long‐spined sea urchin *Diadema antillarum* in the Caribbean (Hewson et al. 2023). This scuticociliate has since been grown in xenic culture as isolate FWC2 (Hewson et al. 2023; Ritchie et al. 2024) and identified to be the causative pathogen of other urchin mass mortality events in various geographic regions (Zirler et al. 2023; Ritchie et al. 2024; Roth et al. 2024). Despite the availability of cultured isolates, the genetic repertoire of isolate FWC2 has not yet been characterized. Using a combination of short‐read (Illumina) and long‐read (Oxford Nanopore Technology; ONT) sequencing technologies, we sequenced the metagenomic content of *Philaster* sp. isolate FWC2 to obtain information on its genetic features, including its haploid genome size, nuclear ribosomal operon, and mitochondrial genome (mitogenome).

## 2 Methods

### 2.1 DNA Extraction

*Philaster* sp. isolate FWC2 (available upon request to MB) was grown twice in eight (first round) or nine (second round) 50 mL tubes containing ~45 mL of autoclaved seawater, an autoclaved grain of rice, 10% yeast extract, and 0.2 μm filtered sea urchin homogenate (Hewson et al. 2023). Three 2 μL aliquots from each tube were counted daily under a dissecting microscope (Zeiss Group) to determine the cells per μL and estimate total cell counts. Cultures were harvested for DNA extraction after 3–5 days of growth upon reaching > 100,000 cells. Cultures were pre‐filtered through a 70 μm Falcon nylon cell strainer to remove rice particles, then vacuum filtered onto 5 μm Durapore membrane filters (MilliporeSigma) to reduce co‐occurring bacteria and to isolate macronuclear DNA (Kim et al. 2025). Filters were replaced when they clogged. Six and three filters were used in the first and second round of DNA extraction, respectively. Each filter was added to 600 μL of buffer RLT (Qiagen) and vortexed for 1 min. Cell lysis was confirmed by observing a 2 μL aliquot of the supernatant under a dissecting microscope. The supernatant from each round was pooled for DNA extraction using Qiagen's AllPrep DNA/RNA Mini kit. All centrifugation steps were performed at 10,000 x *g* to prevent DNA shearing. The quantity and quality of each DNA sample was assessed with the Qubit DNA high sensitivity assay (Invitrogen), the Nanodrop ND‐1000 spectrophotometer, and the Genomic DNA ScreenTape on the Agilent 4200 Tapestation.

### 2.2 Library Preparation and Sequencing

DNA extracted in the first round was sent to Michigan State University's Research Technology Support Facility for library preparation using ONT's Ligation Sequencing Kit V14 (SQK‐LSK114). The library was sequenced on a FLO‐MIN114 flow cell on the GridION X5 instrument. Basecalling was performed using Dorado v7.2.13 with the high‐accuracy model. DNA extracted in the second round was sent to Seqcenter for library preparation using the tagmentation‐based and PCR‐based Illumina DNA Prep kit and custom 10 bp unique dual indices (Integrated DNA Technologies) with a target insert size of 280 bp. The library was sequenced on Illumina's NovaSeq X Plus 2 × 151 bp platform. Demultiplexing, quality control and adapter trimming were performed with bcl‐convert1 v4.2.4 (Illumina). Raw reads are deposited in the National Center for Biotechnology Information's (NCBI) Sequence Read Archive (SRA) under the BioProject accession PRJNA1345750.

### 2.3 Genome Size Estimation

The genome heterozygosity, repeat content, and size of isolate FWC2 were estimated from Illumina‐sequenced short reads. Raw reads were taxonomically classified by Kraken v2.1.2 (Wood et al. 2019) against Kraken's standard database (version 12/28/2024) containing archaeal, bacterial, viral, plasmid, and human sequences from NCBI RefSeq (O'Leary et al. 2016) and nucleotide sequences of vectors, adapters, linkers, and primers from NCBI's UniVec_Core database (https://www.ncbi.nlm.nih.gov/tools/vecscreen/univec/). Unclassified reads were extracted using the extract_kraken_reads.py script in KrakenTools v1.2 (Lu et al. 2022). *k*‐mers of sizes 18, 21, 24, 27, and 30 were counted in the unclassified reads using the count and histo commands in Jellyfish v2.3 (Marçais and Kingsford 2011). Histogram files of *k*‐mer occurrences were uploaded to the GenomoScope v2.0 server (Ranallo‐Benavidez et al. 2020) using the parameters ploidy = 1 (for haploid genomes) and max_kmercov = 30 (Table S1).

### 2.4 Metagenome Assembly

Illumina‐sequenced reads were trimmed using Trimmomatic v0.39 (Bolger et al. 2014) to remove leading and trailing bases with a quality score of < 3 (LEADING:3 and TRAILING:3), bases with a quality score of < 30 in a 4‐base wide sliding window (SLIDINGWINDOW:4:30), and bases below 50 bp (MINLEN:50). Read quality before and after trimming was assessed using FastQC v0.11.8 (https://www.bioinformatics.babraham.ac.uk/projects/fastqc). Raw ONT reads and trimmed Illumina reads were co‐assembled using hybridSPAdes v3.15.3 (Antipov et al. 2016) implemented on the KBase server (Arkin et al. 2018) and OPERA‐MS v0.9.0 (Bertrand et al. 2019). Raw ONT reads were also assembled using the ‐‐nano‐raw and ‐‐nano‐hq modes of Flye v2.9.3 (Kolmogorov et al. 2019), the default parameters of Raven v1.5.0 (Vaser and Šikić 2021), and Canu v2.2 (Koren et al. 2017) using a preliminary genome size specification of 50 Mbp. Benchmarking sets of Universal Single‐Copy Orthologs (BUSCO) v5.6.1 (Manni et al. 2021) were used to estimate the completeness of the long‐read library and each metagenomic assembly using the alveolata_odb10 and eukaryota_odb10 datasets (Table S2).

### 2.5 Metagenome Annotation

A custom Basic Local Alignment Search Tool (BLAST) database was created from the metagenomic assemblies using the makeblastdb function in NCBI BLAST+ v2.14.0 (Camacho et al. 2009). Reference sequences of the 18S rRNA, 28S rRNA, 5.8S rRNA, and the Internal Transcribed Spacer (ITS) previously amplified from *Philaster* sp. isolate FWC2 (GenBank accessions: PX139012, OP896853, and OP896845) (Hewson et al. 2023), the mitogenome sequences of a related scuticociliate species *Uronema marinum* (GenBank accession: MG272262) (Li et al. 2018) and another ciliate species *Paramecium aurelia* (GenBank accession: X15917) (Pritchard et al. 1990), and the cyclic adenosine monophosphate (cAMP)‐dependent protein kinase A (β‐PKA) protein sequence reported to be upregulated in *Philaster apodigitiformis* during host infection (Zhou et al. 2024) were queried against the custom BLAST database. Matching ribosomal operon sequences and mitogenome sequences were separately aligned using the FFT‐NS‐2 method on the MAFFT v7.5.11 server (Katoh et al. 2019). Each alignment was manually curated in Unipro UGENE v48.1 (Okonechnikov et al. 2012) to obtain a consensus sequence for the nuclear ribosomal operon (GenBank accession: PX460323) and the mitogenome (GenBank accession: PX354982).

### 2.6 Mitogenome Annotation

The mitogenome was annotated by identifying open reading frames (ORFs) using NCBI's ORF finder with genetic code 4 and by comparing its nucleotide and ORF sequences with annotated sequences in the *Uronema marinum* mitogenome (Li et al. 2018) using various programs on the NCBI's BLAST server (Johnson et al. 2008). The mitogenome was visualized using Circos v0.69–8 (Krzywinski et al. 2009) implemented in MitoZ v3.6 (Meng et al. 2019). MEGA v11.0.13 (Tamura et al. 2021) was used to calculate the nucleotide composition and the relative synonymous codon usage (RSCU) values (Sharp and Li 1987) of the mitogenome and other mitogenomes from the scuticociliate species *Uronema marinum* and *Pseudocohnilembus persalinus*. RSCU values were visualized using the ggplot2 R package (Wickham 2016).

### 2.7 Phylogenetic Analysis

Nucleotide sequences of the 18S rRNA gene, ITS1‐5.8S rRNA‐ITS2, and 28S rRNA gene identified in isolate FWC2's nuclear ribosomal operon were compared with sequences from 15 other scuticociliate species and one outgroup species retrieved by blastn searches against the core nucleotide (core_nt) database on NCBI's BLAST server (Johnson et al. 2008) (Table S3). Protein‐coding genes (PCGs) in isolate FWC2's mitogenome were compared with annotations from 26 other Ciliophora mitogenomes retrieved from NCBI's Organelle Genome Resources (Sayers et al. 2021) to identify 18 PCGs shared by 19 mitogenomes (Table S4). Sequences from each gene were separately aligned using the FFT‐NS‐2 method on the MAFFT v7.5.11 server (Katoh et al. 2019). The multiple sequence alignments (MSAs) were concatenated using MEGA v11.0.13 (Tamura et al. 2021) into a nuclear ribosomal MSA and a mitochondrial PCG MSA. gblocks v0.91.1 (Castresana 2000) implemented on the NGPhylogeny.fr server (Lemoine et al. 2019) was used to identify conserved blocks of 2067 bp for the nuclear ribosomal MSA and 2929 aa for the mitochondrial PCG MSA. From each conserved block, a Maximum Likelihood (ML) tree with Shimodaira–Hasegawa‐like aLRT branch support (Guindon et al. 2010) was generated using the default parameters of phyML v1.8.1_1 with Smart Model Selection (SMS) (Lefort et al. 2017) on the NGPhylogeny.fr server (Lemoine et al. 2019). Based on the Aikaike Information Criterion (AIC) (Akaike 1974), the models selected for the nuclear ribosomal MSA and mitochondrial PCG MSA were GTR + G + I and MtZoa+G + F, respectively. Each tree was visualized in FigTree v1.4.4 (https://tree.bio.ed.ac.uk/software/figtree/).

## 3 Results and Discussion

This study provides the first genomic resource of a scuticociliate pathogen, *Philaster* sp. isolate FWC2, within the family Philasteridae. Illumina sequencing generated 493 million 2 × 151 bp reads, with 379 million reads (77%) remaining after trimming. ONT sequencing generated 1.8 million reads with Q ≥ 9, with a *N*_*50*_ value of 14,823 bp and mean length of 8,615 bp. The haploid genome size of isolate FWC2 was estimated from the Illumina‐sequenced reads to be between 122 Mbp (*k* = 18) and 136 Mbp (*k* = 24) (Table S1). This estimate was similar to the 126 Mbp genome size of the marine ciliate *Diophyrs* sp. (Chen et al. 2021), but larger than the 65 Mbp and 75 Mbp genomes of the scuticociliate pathogens *Pseudocohnilembus persalinus* (Xiong et al. 2015) and *Miamiensis avidus* (Kim et al. 2021), respectively. This estimate was also larger than the genome sizes of other ciliate species, which ranged from 42 to 111 Mbp (Kim et al. 2025; Chen et al. 2021; Eisen et al. 2006). Sequenced long‐reads covered 46% of the Alveolata Benchmarking sets of Universal Single‐Copy Orthologs (BUSCOs) (Manni et al. 2021) and 24% of the Eukaryota BUSCOs (Table S2). Hybrid assembly methods produced more complete Alveolata BUSCOs (53%–56%) than long‐read assembly approaches that produced 19%–43% completeness, at the cost of a higher number of contigs and lower *N*_50_ values. Although the number of macronuclei and micronuclei of isolate FWC2 is unknown, the pore size of 5 μm used in this study was previously used to isolate macronuclear DNA from *Miamiensis avidus* (Kim et al. 2025). Therefore, our sequences should primarily represent the macronuclear genome. Nevertheless, further purification of macronuclear DNA (Swart et al. 2013), combined with single‐cell genomics (Chen et al. 2021), will improve the genome completeness of this isolate.

From the metagenomic assemblies, we identified a 4,088 bp nuclear ribosomal operon comprising the 18S SSU rRNA, 5.8S rRNA, ITS, and 28S LSU rRNA. Consistent with the phylogeny of Scuticociliata (Gao et al. 2012), sequences from the family Philasteridae were most closely related to sequences from the family Uronematidae, which includes the genera *Uronema*, *Uronemella*, and *Parauronema* (Gao et al. 2012). Sequences of *Philaster* sp. isolate FWC2 from this study were most closely related to sequences previously amplified from the same cultured isolate (Hewson et al. 2023; Ritchie et al. 2024) (Figure S1 and Table S3). Consistent with previous reports (Hewson et al. 2023; Ritchie et al. 2024; Vilanova‐Cuevas et al. 2025), sequences from isolate FWC2 formed a sister clade with sequences from *Philaster apodigitiformis* and *Philaster sinensis*. The clade containing isolate FWC2 is heretofore designated *Diadema antillarum* scuticociliatosis (DaSc)‐associated *Philaster* clade (DaScPc). DaScPc likely represents a distinct lineage that should be evaluated with detailed morphological analysis, including electron microscopy, protargol staining, and silver nitrate impregnation (Miao et al. 2009).

**FIGURE 1:**
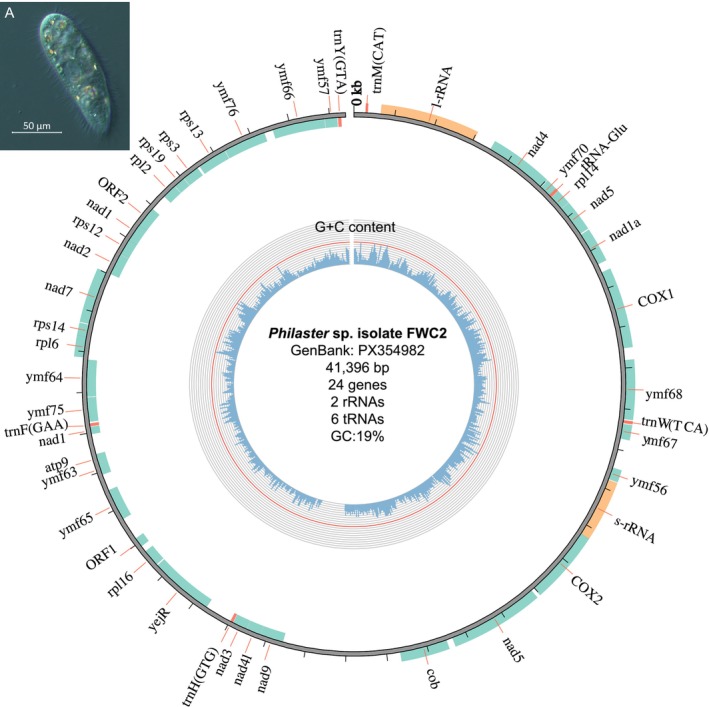
Map of the 41,396 bp mitochondrial genome (mitogenome) of *Philaster* sp. isolate FWC2 (NCBI accession: PX354982), which includes protein‐coding (green), rRNA (orange), and tRNA (red) gene regions. Open reading frames (ORFs) 1 and 2 encode for hypothetical proteins. The red line on the G + C content track delineates the G + C content threshold of 50%. Black tick marks, spaced 1 kbp apart, indicate positions on the mitogenome. Inset A shows a *Philaster* sp. isolate FWC2 individual observed by microscopy.

We also annotated a 41,396 bp mitogenome from the metagenomic data containing 2 rRNA genes, 6 tRNA genes, and 24 PCGs (Figure 1). Most PCGs used the AUG start codon, except for the *rps12* gene encoding the ribosomal protein S12 that used the ATT start codon. PCGs identified included 11 *ymf* genes with unknown functions that are conserved in some ciliates (Burger et al. 2000; Moradian et al. 2007) and two ORFs encoding hypothetical proteins. Phylogenetic analysis of 18 PCGs clustered the *Philaster* sp. isolate FWC2 mitogenome with the mitogenomes of two other scuticociliate species, *Uronema marinum* and *Pseudocohnilembus persalinus*, with 100% branch support (Figure S2 and Table S4). Isolate FWC2 and its closest relative, *Uronema marinum*, had mitogenomes with 19% G + C content, with the former comprising 40% T, 10% C, 41% A, and 9% G and the latter comprising 41% T, 9% C, 40% A, and 10% G. In comparison, the *Pseudocohnilembus persalinus* mitogenome had a higher (23%) G + C content with 40% T, 11% C, 38% A, and 12% G. These three scuticociliate mitogenomes shared similar relative synonymous codon usage (RSCU) values (Figure S3). The most commonly used codons in these mitogenomes (> 2 RSCU) included AGA for arginine and UUA for leucine. In contrast, the least commonly used codons in these mitogenomes (< 0.3 RSCU) included CGC for arginine, CUC for leucine, UUC for phenylalanine, and CCG for proline. UAA was more commonly used as the stop codon (1.5–1.6 RSCU) compared to UAG (0.4–0.5 RSCU) in these mitogenomes.

The *β‐PKA* gene was upregulated in *Philaster apodigitiformis* during infection of the guppy *Poecilia reticulata* and hypothesized to contribute to pathogenesis (Zhou et al. 2024). From isolate FWC2's metagenome, we identified a 83 aa protein homolog that shared 83% identity with the *Philaster apodigitiformis* β‐PKA protein (Figure S4). Future transcriptomic and molecular investigations will provide valuable insights into the *β‐PKA* gene expression and physiopathology of DaScPc.

## Author Contributions

S.J.L.: funding acquisition, investigation, methodology, data curation, formal analysis, software, supervision, visualization, writing – original draft. M.B.: conceptualization, funding acquisition, investigation, methodology, project administration, resources, supervision, validation, writing – review and editing.

## Funding

This work was supported by the University of South Florida. National Science Foundation, OCE‐ 2527605.

## Consent

All authors consent to the publication of this article.

## Conflicts of Interest

The authors declare no conflicts of interest.

## Supporting information


**Data S1:** jeu70065‐sup‐0001‐Supinfo.zip.

## Data Availability

The data that support the findings of this study are openly available in NCBI at https://www.ncbi.nlm.nih.gov/bioproject/PRJNA1345750/ under the accession numbers PX460323, PX354982, and SRX31190121–SRX31190122.
