# Supplementary material for: Genetic Features of the Scuticociliate Pathogen Philaster sp. Isolate FWC2 That Causes Sea Urchin Mass Mortality
Source: J Eukaryot Microbiol. 2026 Feb 2;73(2):e70065. doi: 10.1111/jeu.70065 (PMC12865139; doi:10.1111/jeu.70065)
Supplement: Supplementary file 1 — Data S1: jeu70065‐sup‐0001‐Supinfo.zip. [file JEU-73-e70065-s001.zip › jeu70065-sup-0002-FigureS2@FigS2-mt-phylogeny.pdf]

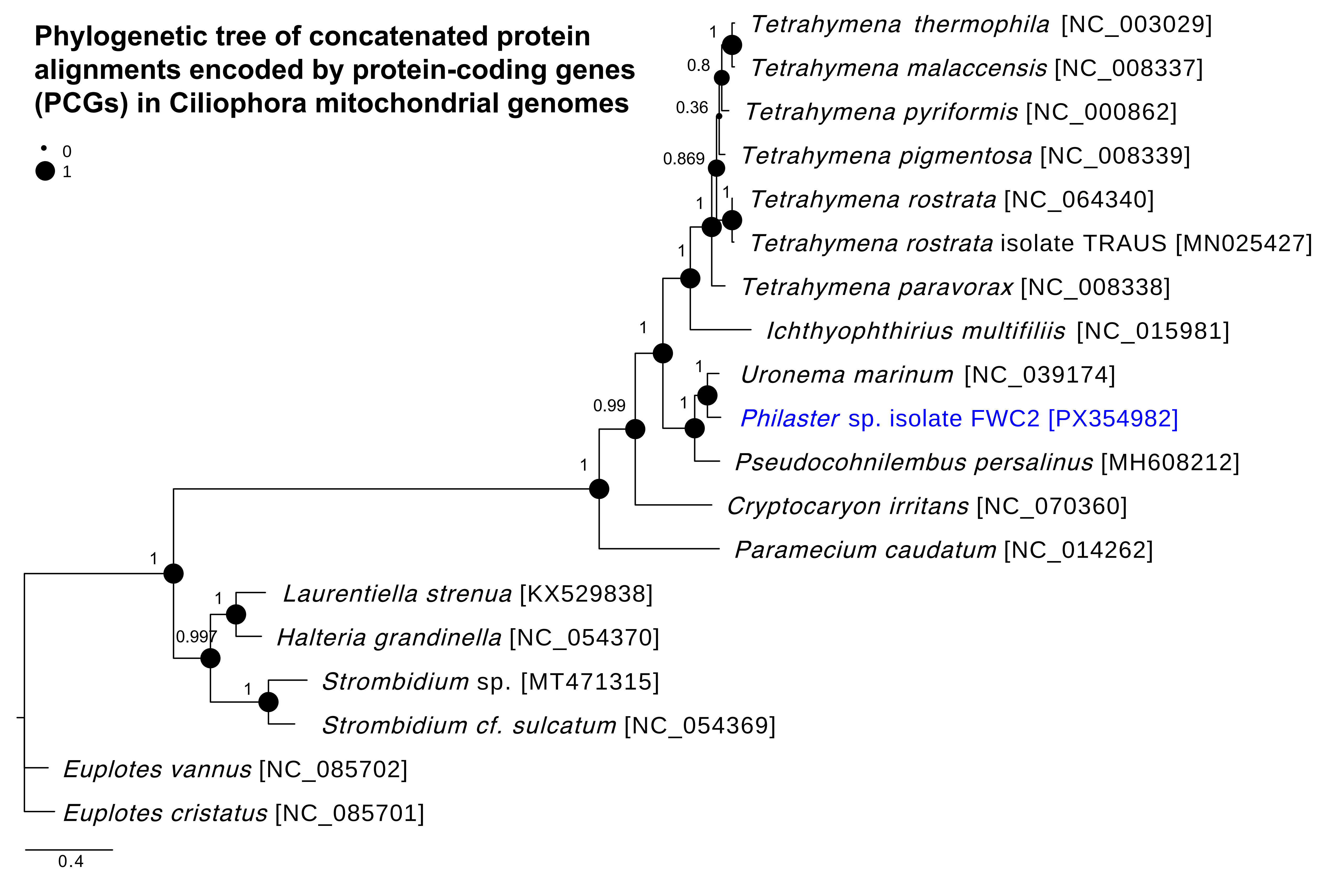

Phylogenetic tree of concatenated protein alignments encoded by protein-coding genes (PCGs) in Ciliophora mitochondrial genomes
0
1
Tetrahymena thermophila [NC_003029]
Tetrahymena malaccensis [NC_008337]
Tetrahymena pyriformis [NC_000862]
Tetrahymena pigmentosa [NC_008339]
Tetrahymena rostrata [NC_064340]
Tetrahymena rostrata isolate TRAUS [MN025427]
Tetrahymena paravorax [NC_008338]
Ichthyophthirius multifiliis [NC_015981]
Uronema marinum [NC_039174]
Philaster sp. isolate FWC2 [PX354982]
Pseudocohnilembus persalinus [MH608212]
Cryptocaryon irritans [NC_070360]
Paramecium caudatum [NC_014262]
Laurentiella strenua [KX529838]
Halteria grandinella [NC_054370]
Strombidium sp. [MT471315]
Strombidium cf. sulcatum [NC_054369]
Euplotes vannus [NC_085702]
Euplotes cristatus [NC_085701]
1
0.8
0.36
0.869
1
1
1
1
1
1
0.99
1
1
1
0.997
1
0.4
