# Supplementary material for: Genetic Features of the Scuticociliate Pathogen Philaster sp. Isolate FWC2 That Causes Sea Urchin Mass Mortality
Source: J Eukaryot Microbiol. 2026 Feb 2;73(2):e70065. doi: 10.1111/jeu.70065 (PMC12865139; doi:10.1111/jeu.70065)

Comparisons of relative synonymous codon usage (RSCU) values across scuticociliate mitogenomes

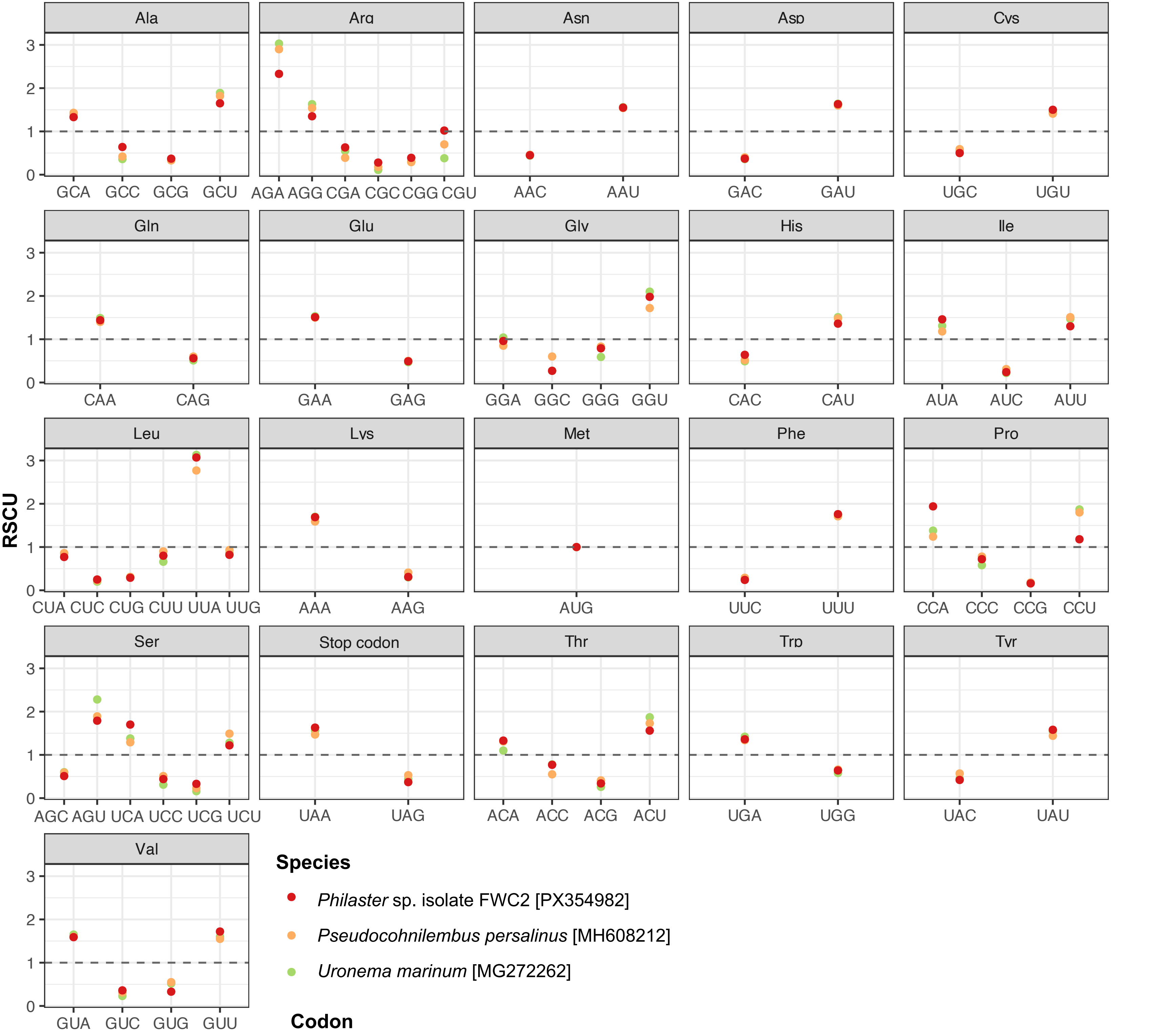

Supplement: Supplementary file 1 — Data S1: jeu70065‐sup‐0001‐Supinfo.zip. [file JEU-73-e70065-s001.zip › jeu70065-sup-0003-FigureS3@FigS3-RSCU.pdf]
