# Supplementary material for: Genetic Features of the Scuticociliate Pathogen Philaster sp. Isolate FWC2 That Causes Sea Urchin Mass Mortality
Source: J Eukaryot Microbiol. 2026 Feb 2;73(2):e70065. doi: 10.1111/jeu.70065 (PMC12865139; doi:10.1111/jeu.70065)
Supplement: Supplementary file 1 — Data S1: jeu70065‐sup‐0001‐Supinfo.zip. [file JEU-73-e70065-s001.zip › Supp.docx]

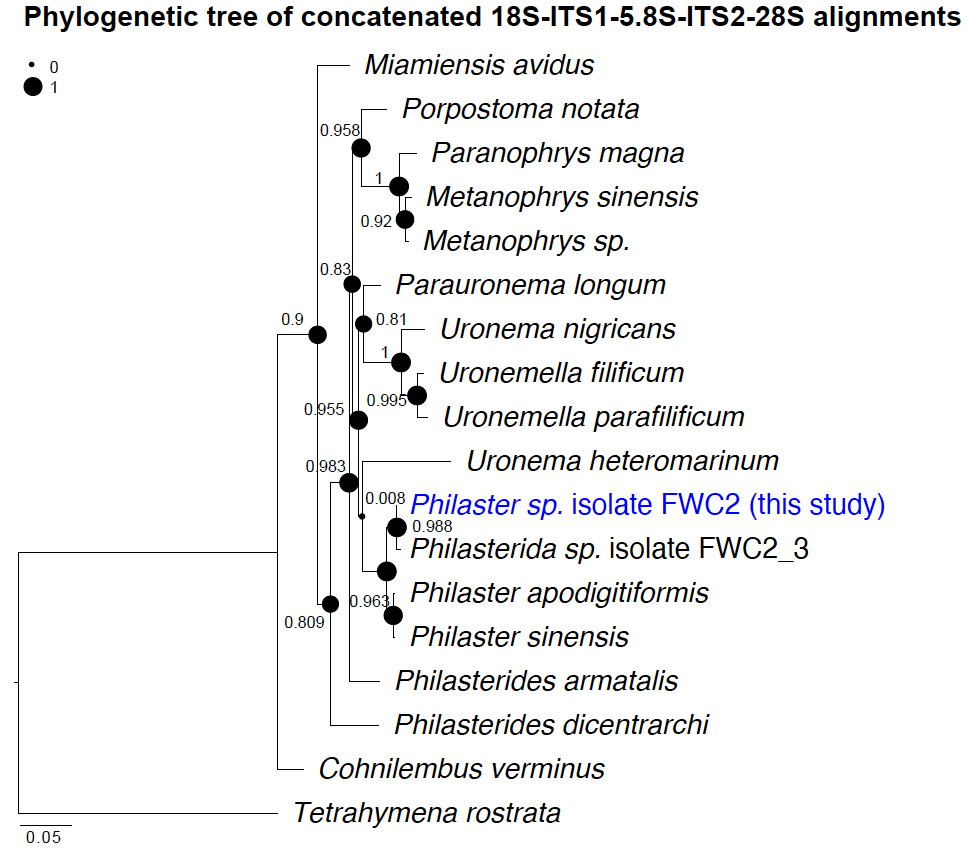


**Figure S1.** Maximum likelihood phylogenetic tree generated from the concatenated 16S rRNA, Internal Transcribed Spacer (ITS) 1, 5.8S rRNA, ITS2, and 28S rRNA nucleotide sequence alignments assembled from the *Philaster* sp. isolate FWC2 metagenomic libraries (blue text) in relation to sequences from other species within the subclass Scuticociliata. Node sizes and labels indicate Shimodaira–Hasegawa-like aLRT branch support values. Branch lengths indicate the number of substitutions per site. The outgroup species used was *Tetrahymena rostrata* from the subclass Hymenostomatia. Accession numbers of these sequences are provided in **Table S3**.

**
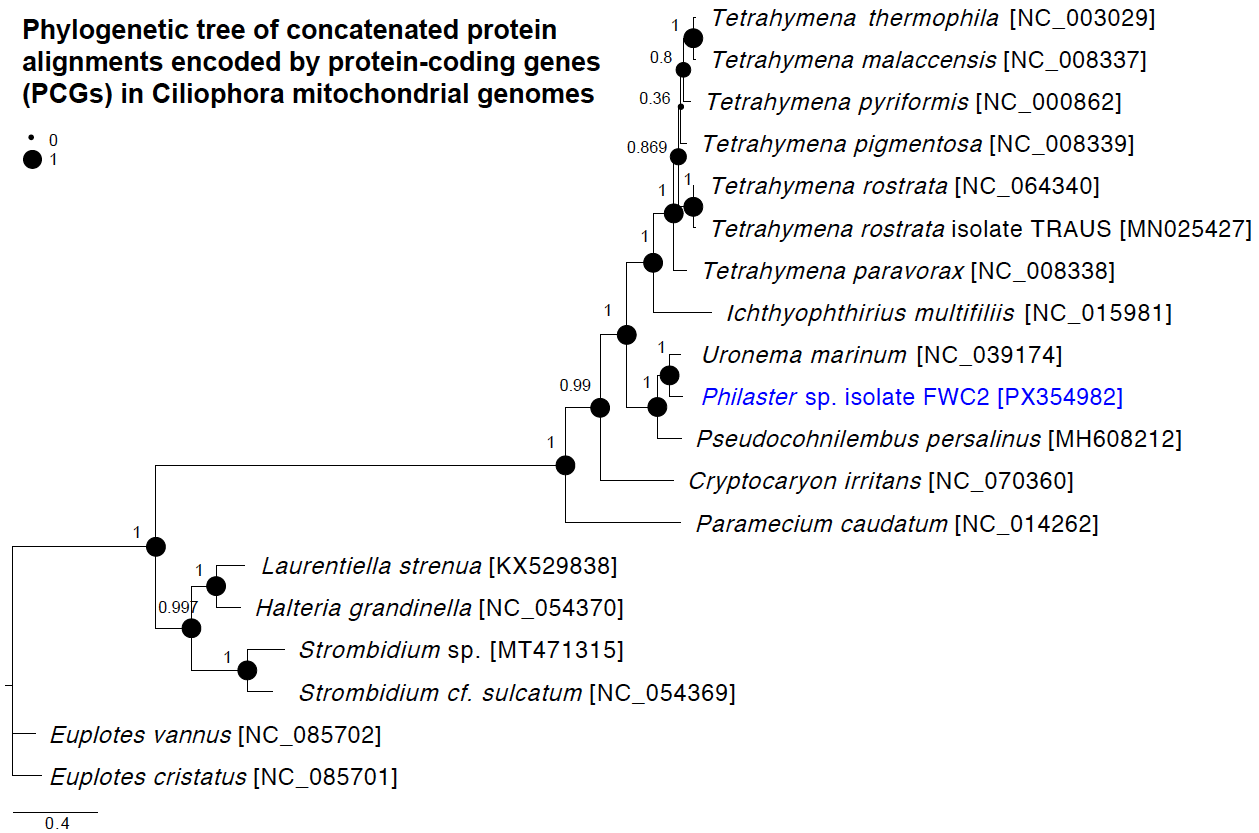
**

**Figure S2.** Maximum likelihood phylogenetic tree generated from the concatenated protein sequence alignments of 18 PCGs shared by the *Philaster* sp. isolate FWC2 mitochondrial genome (mitogenome; blue text) and 18 other Ciliophora mitogenomes. Accession numbers of these sequences are provided in square brackets. Node sizes and labels indicate Shimodaira–Hasegawa-like aLRT branch support values. Branch lengths indicate the number of substitutions per site. The outgroup species used was *Euplotes cristatus* from the class Spirotrichea**.**

**
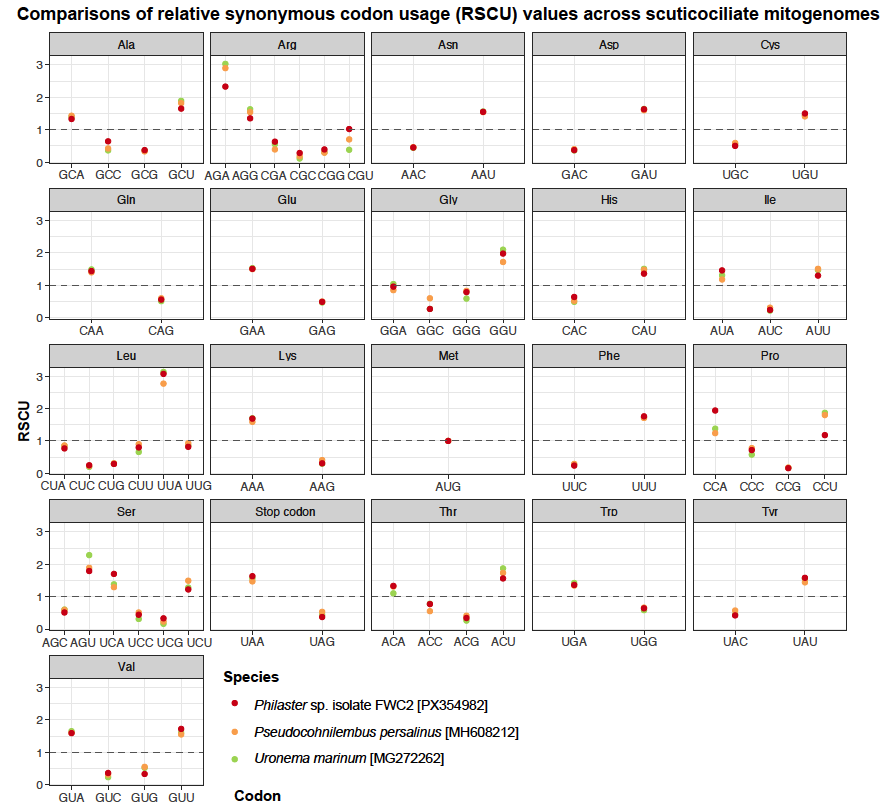
**

**Figure S3.** Comparisons of relative synonymous codon usage (RSCU) values between the *Philaster* sp. isolate FWC2 mitochondrial genome (mitogenome) and two other scuticociliate mitogenomes. The dashed horizontal line on each plot delineates the RSCU threshold of 1. Accession numbers of these mitogenome sequences are provided in square brackets in the legend.

**β-PKA** 1 MFEYLHNKNIVYRDLKPENILIGSDGYLKLTDFGFAKYCDSRTYTLCGTPEYLAPEILLNKGHGKPVDWWCLGILIYEMLAGIDPF 86

MFEYLH+KNIVYRDLKPENILI +DGYLKLTDFGFAKYCDSRTYTLCGTPEYLAPEILLNKGHGKPVDW+ I I + +L G F

**FWC2** 1 MFEYLHSKNIVYRDLKPENILIAADGYLKLTDFGFAKYCDSRTYTLCGTPEYLAPEILLNKGHGKPVDWY - - - IYIF ILLRGFTFF 86

**Figure S4.** tblastn alignment of the 196 aa β-PKA protein sequence in *Philaster apodigitiformis* and its homolog in the *Philaster* sp. isolate FWC2 metagenome. The 86 aa local sequence alignment shared 83% identity and 88% similarity with an alignment score of 148 bits (378) and expect value (e-value) of 6x10^-40^.

**Supplemental Data**

**Table S1.** Genome properties of *Philaster* sp. isolate FWC2 predicted by GenomoScope 2.0. Abbreviations: % unique, percent of the genome that is unique (not repetitive); kcov, mean *k*-mer coverage for heterozygous bases; dup, average rate of read duplications.

|  | *k*=18 | | *k*=21 | | *k*=24 | | *k*=27 | | *k*=30 | |
| --- | --- | --- | --- | --- | --- | --- | --- | --- | --- | --- |
|  | min | max | min | max | min | max | min | max | min | max |
| **Homozygous (aa)** | 100% | 100% | 100% | 100% | 100% | 100% | 100% | 100% | 100% | 100% |
| **Genome haploid length (bp)** | 116,944,893 | 122,002,673 | 130,817,078 | 136,171,038 | 130,369,208 | 136,272,828 | 125,779,849 | 131,897,374 | 120,959,747 | 126,897,930 |
| **Genome repeat length (bp)** | 22,478,617 | 23,450,800 | 24,791,104 | 25,805,732 | 24,686,749 | 25,804,660 | 24,148,509 | 25,323,014 | 23,750,466 | 24,916,430 |
| **Genome unique length (bp)** | 94,466,276 | 98,551,873 | 106,025,973 | 110,365,307 | 105,682,459 | 110,468,168 | 101,631,340 | 106,574,360 | 97,209,280 | 101,981,500 |
| **% unique** | 80.78 | 80.78 | 81.05 | 81.05 | 81.06 | 81.06 | 80.80 | 80.80 | 80.36 | 80.36 |
| **Model fit** | 97.97% | 97.97% | 97.60% | 97.60% | 97.26% | 97.26% | 96.89% | 96.89% | 96.53% | 96.53% |
| **Read error rate** | 2.30% | 2.30% | 2.20% | 2.20% | 2.10% | 2.10% | 2.02% | 2.02% | 1.94% | 1.94% |
| **kcov** | 14.9 | 14.9 | 14.6 | 14.6 | 14.5 | 14.5 | 14.4 | 14.4 | 14.4 | 14.4 |
| **dup** | 1.31 | 1.31 | 1.19 | 1.19 | 1.09 | 1.09 | 1.01 | 1.01 | 1.94 | 1.94 |

**Table S2.** Features of the long-read library generated by Oxford Nanopore Technology (ONT) sequencing and the metagenomes assembled by various methods in this study. Completeness metrics were estimated by comparing sequences against Benchmarking sets of Universal Single-Copy Orthologs (BUSCO) reference lineage datasets.

| **Metagenomic library/assembly** | **BUSCO dataset** | **Complete (%)** | **Complete and single-copy (%)** | **Complete and duplicated (%)** | **Fragmented (%)** | **Missing (%)** | **Number of sequences** | **Total length (Gbp)** | **N50 (kbp)** |
| --- | --- | --- | --- | --- | --- | --- | --- | --- | --- |
| **Raw reads** |  |  |  |  |  |  |  |  |  |
| ONT reads | alveolata_odb10 | 46.20% | 22.22% | 23.98% | 12.28% | 41.52% | 1,783,696 | 15.37 | 14.62 |
| ONT reads | eukaryota_odb10 | 23.92% | 9.80% | 14.12% | 11.37% | 64.71% | 1,783,696 | 15.37 | 14.62 |
|  |  |  |  |  |  |  |  |  |  |
| **Long-read assemblies** |  |  |  |  |  |  |  |  |  |
| Flye (nanoraw mode) | alveolata_odb10 | 42.69% | 30.99% | 11.70% | 12.87% | 44.44% | 4,335 | 0.27 | 103 |
| Flye (nanoraw mode) | eukaryota_odb10 | 39.61% | 31.76% | 7.84% | 7.06% | 53.33% | 4,335 | 0.27 | 103 |
| Flye (nanohq mode) | alveolata_odb10 | 40.94% | 29.24% | 11.70% | 12.87% | 46.20% | 4,322 | 0.27 | 103 |
| Flye (nanohq mode) | eukaryota_odb10 | 23.92% | 14.90% | 9.02% | 7.84% | 68.24% | 4,322 | 0.27 | 103 |
| Raven | alveolata_odb10 | 18.71% | 5.85% | 12.87% | 3.51% | 77.78% | 1,572 | 0.11 | 1000 |
| Raven | eukaryota_odb10 | 14.51% | 7.84% | 6.67% | 5.49% | 80.00% | 1,572 | 0.11 | 1000 |
| Canu | alveolata_odb10 | 22.81% | 11.11% | 11.70% | 5.26% | 71.93% | 6,168 | 0.13 | 126 |
| Canu | eukaryota_odb10 | 14.51% | 7.84% | 6.67% | 5.49% | 80.00% | 6,168 | 0.13 | 126 |
|  |  |  |  |  |  |  |  |  |  |
| **Hybrid assemblies** |  |  |  |  |  |  |  |  |  |
| OPERA-MS | alveolata_odb10 | 53.22% | 40.35% | 12.87% | 13.45% | 33.33% | 172,145 | 0.51 | 29 |
| OPERA-MS | eukaryota_odb10 | 27.45% | 15.29% | 12.16% | 10.59% | 61.96% | 172,145 | 0.51 | 29 |
| HybridSPAdes | alveolata_odb10 | 56.14% | 44.44% | 11.70% | 11.11% | 32.75% | 58,363 | 0.45 | 36 |
| HybridSPAdes | eukaryota_odb10 | 27.45% | 14.90% | 12.55% | 11.37% | 61.18% | 58,363 | 0.45 | 36 |

**Table S3.** GenBank accession numbers for the scuticociliate species and outgroup species (*Tetrahymena rostrata*) used for phylogenetic analysis. The nuclear ribosomal operon sequence assembled from this study is highlighted in bold.

| **Species** | **18S rRNA** | **5.8S rRNA and ITS** | **28S rRNA** |
| --- | --- | --- | --- |
| ***Philaster* sp. isolate FWC2 (this study)** | **PX460323** | **PX460323** | **PX460323** |
| *Philasterida* sp. isolate FWC2_3 | OP896853 | OP896845 | PP784536 |
| *Philaster apodigitiformis* | FJ648350 | JN885097 | JN885119 |
| *Philasterides armatalis* strain GF2008062601 | FJ848877 | JN885098 | JN885120 |
| *Philaster sinensis* isolate PXM2011102701 | KJ815049 | PP852879 | PX139012 |
| *Philasterides dicentrarchi* isolate I1 | MK002746 | MK002746 | MK002746 |
| *Cohnilembus verminus* isolate FXP2009051101 | HM236339 | JN885093 | JN885111 |
| *Metanophrys sinensis* isolate FXP2009052901 | JN885089 | JN885105 | JN885116 |
| *Metanophrys* sp. isolate ZRL2022050202 | HM236336 | JN885092 | JN885114 |
| *Miamiensis avidus* isolate FXP2009050602 | JN885091 | JN885095 | JN885115 |
| *Paranophrys magna* isolate FXP2009052803 | HM236335 | JN885099 | JN885121 |
| *Parauronema longum* isolate FXP2009031501 | OR287093 | OR282527 | OR287092 |
| *Porpostoma notata* isolate FXP2009050601 | HM236338 | JN885096 | JN885118 |
| *Uronema heteromarinum* | FJ870100 | JN885101 | JN885123 |
| *Uronema nigricans* | JF973324 | MW785175 | PP784543 |
| *Uronemella filificum* isolate LMJ2016040402 | MG581967 | PP852882 | PP784542 |
| *Uronemella parafilificum* isolate FXP2009053001 | HM236337 | JN885103 | JN885127 |
| *Tetrahymena rostrata* strain TRAUS (outgroup) | MN158348 | MN158348 | MN158348 |

**Table S4.** List of the protein-coding genes (PCGs) annotated in the *Philaster* sp. isolate FWC2 mitogenome and their corresponding NCBI Protein accession numbers. PCGs used for phylogenetic analysis are highlighted in bold.

| **Gene** | ***Philaster* sp. isolate FWC2** |
| --- | --- |
| ***cox1*** | **YAJ48230** |
| ***cox2*** | **YAJ48234** |
| ***atp9*** | **YAJ48244** |
| ***nad10*** | **YAJ48253** |
| ***nad7*** | **YAJ48250** |
| ***nad1_a*** | **YAJ48228** |
| ***nad3*** | **YAJ48239** |
| ***nad4*** | **YAJ48225** |
| ***nad9*** | **YAJ48237** |
| ***nad5*** | **YAJ48235** |
| ***rpl14*** | **YAJ48227** |
| ***rpl16*** | **YAJ48241** |
| ***rpl2*** | **YAJ48255** |
| ***rps12*** | **YAJ48252** |
| ***rps13*** | **YAJ48258** |
| ***rps14*** | **YAJ48249** |
| ***rps19*** | **YAJ48256** |
| ***rps3*** | **YAJ48257** |
| *nad1_b* | YAJ48245 |
| *nad2* | YAJ48251 |
| *nad4l* | YAJ48225 |
| *nad6* | YAJ48229 |
| *cob* | YAJ48236 |
| *rpl6* | YAJ48248 |
| *yejR* | YAJ48240 |
| *ymf57* | YAJ48261 |
| *ymf63* | YAJ48243 |
| *ymf64* | YAJ48247 |
| *ymf65* | YAJ48242 |
| *ymf66* | YAJ48260 |
| *ymf67* | YAJ48232 |
| *ymf68* | YAJ48231 |
| *ymf70* | YAJ48226 |
| *ymf75* | YAJ48246 |
