# Supplementary figures and images for: Genetic Features of the Scuticociliate Pathogen Philaster sp. Isolate FWC2 That Causes Sea Urchin Mass Mortality
Source: J Eukaryot Microbiol. 2026 Feb 2;73(2):e70065. doi: 10.1111/jeu.70065 (PMC12865139; doi:10.1111/jeu.70065)

# Phylogenetic tree of concatenated 18S-ITS1-5.8S-ITS2-28S alignments

• 0  
● 1

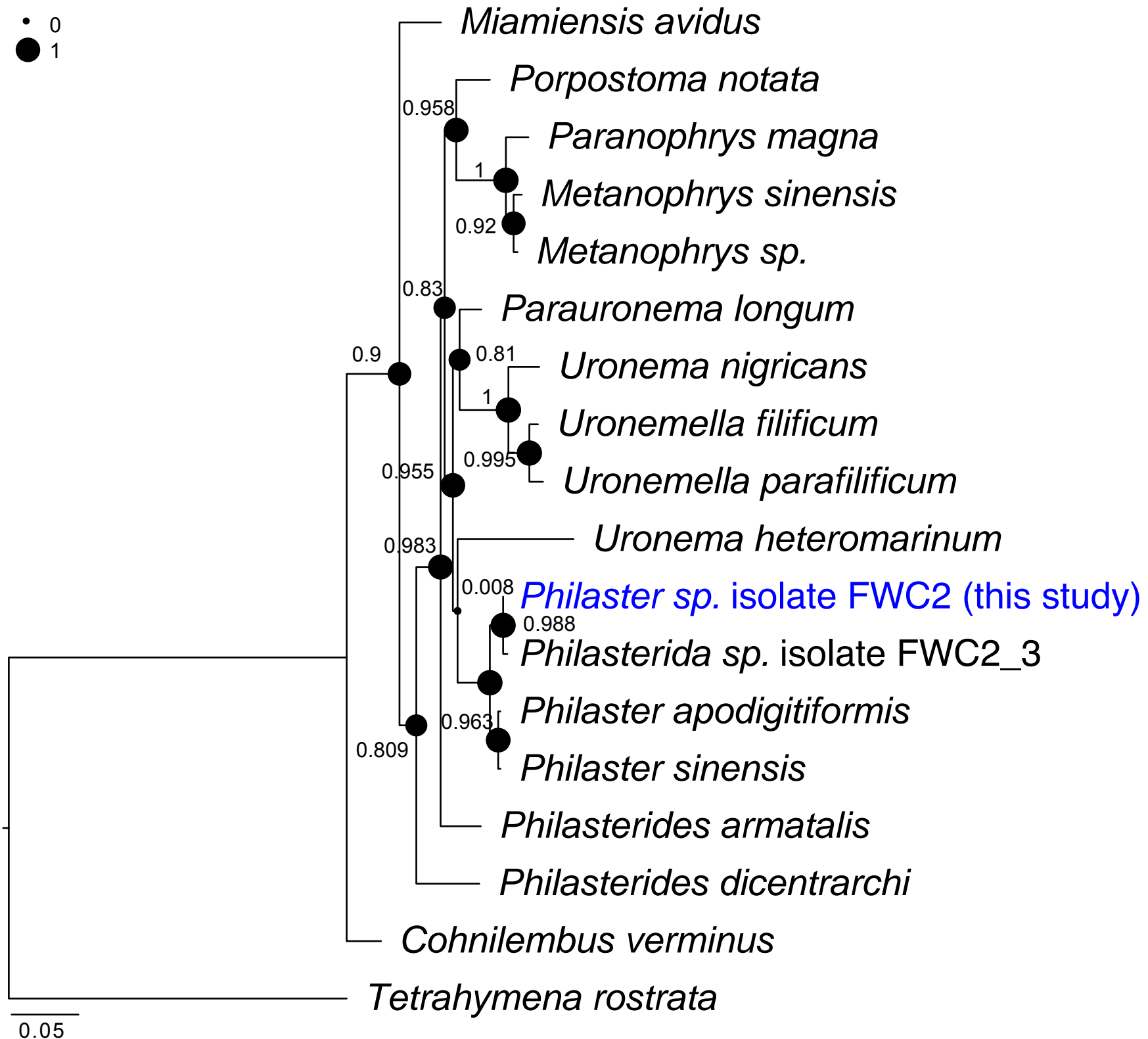

Supplement: Supplementary file 1 — Data S1: jeu70065‐sup‐0001‐Supinfo.zip. [file JEU-73-e70065-s001.zip › jeu70065-sup-0001-FigureS1@FigS1-rRNA-tree.pdf]
